# Genetic Manipulation of Caveolin-1 in a Transgenic Mouse Model of Aortic Root Aneurysm: Sex-Dependent Effects on Endothelial and Smooth Muscle Function

**DOI:** 10.3390/ijms252312702

**Published:** 2024-11-26

**Authors:** Tala Curry-Koski, Brikena Gusek, Ross M. Potter, T. Bucky Jones, Raechel Dickman, Nathan Johnson, John N. Stallone, Roshanak Rahimian, Johana Vallejo-Elias, Mitra Esfandiarei

**Affiliations:** 1 Biomedical Sciences Program, College of Graduate Studies, Midwestern University, Glendale, AZ 85308, USAbgusek@midwestern.edu (B.G.);; 2 Department of Physiology, College of Graduate Studies, Midwestern University, Glendale, AZ 85308, USA; 3 Department of Anatomy, College of Graduate Studies, Midwestern University, Glendale, AZ 85308, USA; 4 Department of Veterinary Physiology & Pharmacology, Texas A&M University, College Station, TX 77843, USA; 5 Department of Pharmaceutical Sciences, Thomas J. Long School of Pharmacy, University of the Pacific, Stockton, CA 95211, USA; 6 Department of Anesthesiology, Pharmacology & Therapeutics, University of British Columbia, Vancouver, BC V6T 2A1, Canada; 7 Department of Basic Medical Sciences, College of Medicine, University of Arizona, Phoenix, AZ 85004, USA

**Keywords:** Marfan syndrome, aortic aneurysm, caveolin-1, smooth muscle, endothelium

## Abstract

Marfan syndrome (MFS) is a systemic connective tissue disorder stemming from mutations in the gene encoding Fibrillin-1 (Fbn1), a key extracellular matrix glycoprotein. This condition manifests with various clinical features, the most critical of which is the formation of aortic root aneurysms. Reduced nitric oxide (NO) production due to diminished endothelial nitric oxide synthase (eNOS) activity has been linked to MFS aortic aneurysm pathology. Caveolin-1 (Cav1), a structural protein of plasma membrane caveolae, is known to inhibit eNOS activity, suggesting its involvement in MFS aneurysm progression by modulating NO levels. In this study, we examined the role of Cav1 in aortic smooth muscle and endothelial function, aortic wall elasticity, and wall strength in male and female MFS mice (*FBN1^+/Cys1041Gly^*) by generating developing Cav1-deficient MFS mice (MFS/*Cav1*KO). Our findings reveal that *Cav1* ablation leads to a pronounced reduction in aortic smooth muscle contraction in response to phenylephrine, attributable to an increase in NO production in the aortic wall. Furthermore, we observed enhanced aortic relaxation responses to acetylcholine in MFS/*Cav1*KO mice, further underscoring Cav1’s inhibitory impact on NO synthesis within the aorta. Notably, van Gieson staining and chamber myography analyses showed improved elastin fiber structure and wall strength in male MFS/*Cav1KO* mice, whereas these effects were absent in female counterparts. Cav1’s regulatory influence on aortic root aneurysm development in MFS through NO-mediated modulation of smooth muscle and endothelial function, with notable sex-dependent variations.

## 1. Introduction

Marfan syndrome (MFS) is the most common monogenetic autosomal dominant disorder of connective tissue. MFS is caused by mutations in the gene encoding for the large extracellular matrix (ECM) glycoprotein fibrillin-1 (Fbn1). MFS has an estimated prevalence of 1 in 3000–5000 with no biological sex or ethnic bias [1,2]. Fbn1 is one of the major constituents of extracellular microfibrils found in connective tissues that provides scaffolding support for the formation and maturation of elastin fibers, thus supporting the integrity and elasticity of connective tissues [3,4]. In addition to its structural role, Fbn1 can regulate multiple signaling molecules within the ECM, particularly the activity of the cytokine transforming growth factor beta (TGF-β) through its ability to sequester the latent form of TGF-β within the ECM [5,6,7]. The sequestering function of Fbn1 is achieved by binding to latent TGF-β binding proteins (LTBPs) that are essential for the storage and/or timely release of latent TGF-β complexes [5,6,7]. Mutations in the *Fbn1* gene can cause deficiencies in the functional and structural capacities of microfibrils and subsequent increases in the bioavailable form of TGF-β, leading to the over-activation of the TGF-β downstream signaling pathway [8,9].

Studies on MFS patients and the experimental mouse model have shown that the progression of aortic root aneurysms is also associated with endothelial dysfunction, as evidenced by a significant decrease in endothelial nitric oxide synthase (eNOS) activity within the aortic wall [10,11,12]. In our previous report, we were able to show that the overexpression of a constitutively active form of eNOS could block the development of aortic root aneurysms in MFS mice [13].

In the context of vascular biology, eNOS activity is regulated by a complex network of multiple signaling pathways at both extracellular and intracellular levels, involving a long list of multiple stimuli and signaling cascades including shear stress [14], phosphoinositide 3-kinase/Akt [15], bradykinin [16], calcium–calmodulin [17], acetylcholine/G protein-coupled receptors [18], vascular endothelial growth factor (VEGF) [19], estrogen receptors [20], cyclic AMP [21], and caveolin-1 (Cav1) [22,23,24].

Cav1 protein is the main structural component of caveolae on the plasma membrane of all cell types. It is particularly expressed in endothelial cells, smooth muscle cells, skeletal muscle cells, and adipocytes [25,26]. Caveolae are invaginations of lipid rafts, which are highly dense regions of the plasma membrane enriched in proteins, cholesterol, and sphingolipids [27]. The shape and density of these structures allow for many interactions between various signaling molecules and membrane receptors [28,29]. As the main protein in caveolae, Cav1 plays a key role in multiple cellular processes and signaling involved in inflammation, cell migration, cell differentiation, vesicular trafficking, lipid homeostasis, and stress responses. In the vascular wall, Cav1 plays multiple regulatory roles that include controlling eNOS activity in the endothelial cells [24,30,31], regulating the proliferation and migration of vascular smooth muscle cells (SMCs) [32,33], facilitating cholesterol transport and homeostasis in adipose tissue [34,35], and modulating the activity of matrix metalloproteinases (MMPs) involved in wall remodeling [36,37]. In the endothelium, Cav1 negatively regulates (attenuates) eNOS through direct binding. Through the inhibitory effects on eNOS function, Cav1 plays a critical role in regulating vascular tone and blood pressure [38]. Previous studies have shown that disruption of Cav1 expression results in dysregulated NO production, leading to hypertension and atherosclerosis [7,39,40,41].

It is well understood that MFS aortic root aneurysms are associated with endothelial dysfunction and a significant decrease in eNOS phosphorylation and activity [11,13]. Recent studies have also expanded the understanding of vascular pathology in MFS, highlighting processes such as endothelial-to-mesenchymal transition (EndoMT) and SMC de-differentiation, both of which can further contribute to the remodeling and instability of the aortic wall and endothelial dysfunction [42]. Cav-1 also influences SMC phenotype by modulating pathways involved in cellular differentiation, where the loss of Cav-1 is associated with the increased de-differentiation of SMCs, promoting a shift toward a synthetic, less contractile state [33]. Considering the important role of Cav1 in negatively regulating eNOS function in endothelial cells, and its direct functional impact on endothelial and SMCs function, we designed a study to investigate the effects of Cav1 on aortic wall endothelium and SMCs in a mouse model of MFS-associated aortic root aneurysms (*FBN1^+/^^Cys1041Gly^*) by generating male and female MFS mice lacking the *Cav1* gene (MFS/*Cav1*KO). In this study, we test the hypothesis that *Cav1* deletion in MFS mice will lead to reduced aortic root growth, increased endothelium-mediated vasorelaxation, decreased aortic wall elastin fragmentation, and improved aortic wall strength. To test the hypothesis, we have measured the impact of *Cav1* deletion on endothelium-dependent vasorelaxation, aortic diameter, SMCs contraction, and aortic wall structural integrity and strength in both male and female MFS mice using the small vessel chamber myography. We also assessed aortic wall elastin structural integrity in the cross sections of the ascending aorta of MFS mice using van Gieson elastin staining.

## 2. Results

### 2.1. Genetic Cav1 Deletion Disrupts the Formation of Caveolae Invagination in the Aortic Wall

**Figure 1:**
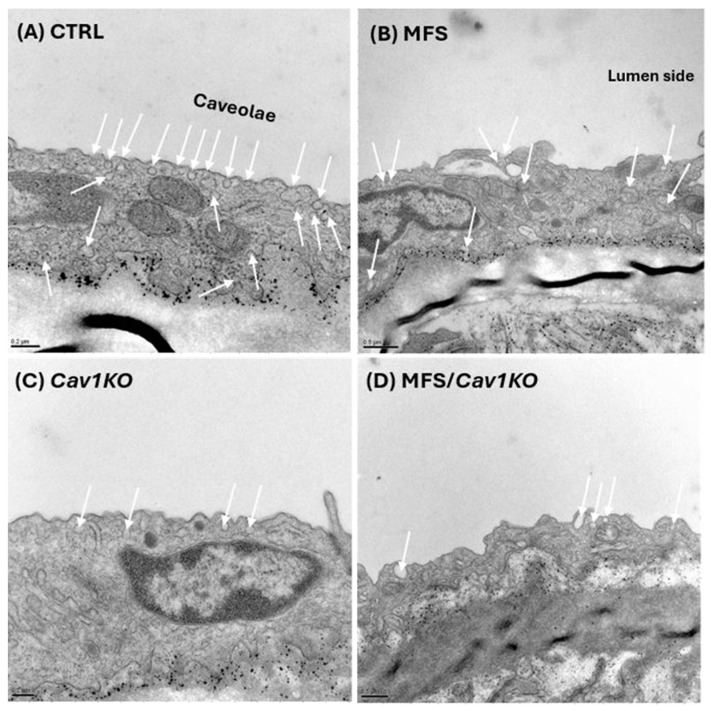
Genetic *Cav1* deletion disrupts the formation of caveolae invagination in the aortic wall. Representative transition electron microscopy images of 9-month-old aortic sections in CTRL (**A**), MFS (**B**), *Cav1KO* (**C**), and MFS/*Cav1KO* (**D**) mice visualize the structure of caveolae invaginations in the aortic wall and shows that deletion of *Cav1* gene impacts the structural integrity and membrane distribution of caveolae.

In addition to confirming the deletion of the *Cav1* gene in mice using PCR, we verified that the ablation of *Cav1* in both control and MFS mice had the anticipated effects on the structure of caveolae in the aortic wall. Through observational and qualitative assessments via electron microscopy (EM) images, we found that the deletion of *Cav1* disrupted the formation of caveolae invaginations on the cell membrane (Figure 1). In CTRL mice aorta, abundant and well-formed caveolae invaginations are clearly visible (white arrows), characterized by flask-shaped membrane invaginations approximately 50–100 nm in diameter (Figure 1A, white arrows). The aortic sections isolated from MFS mice display a reduction in the number of open invaginations (caveolae) on the membrane of endothelial cells (Figure 1B). Likewise, aortic rings from *Cav1* knockout mice (CTRL/*Cav1KO* and MFS/*Cav1KO*) exhibit a reduction in the number of visible caveolae invaginations on the membrane surface (Figure 1C,D). The plasma membrane appeared mostly smooth and devoid of the characteristic invaginated structures seen in the CTRL mice’s aortic sections. This disruption suggests that Cav1 protein plays a critical role in the biogenesis and maintenance of caveolae on cell membranes, and the absence of Cav1 likely impairs the assembly of all associated proteins necessary for caveolae invagination on the membrane surface.

### 2.2. Cav1 Deletion Does Not Affect the Progression of Aortic Root Growth in MFS Mice

**Figure 2:**
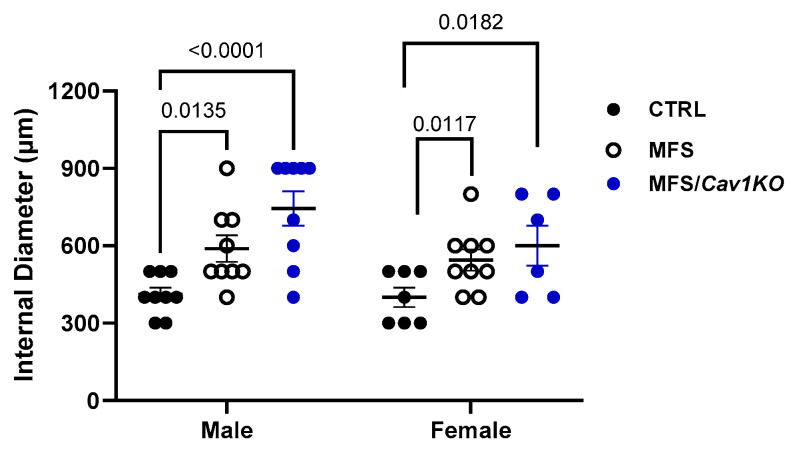
*Cav1* deletion does not affect the progression of aortic root growth in MFS mice. Presented bar graphs showcase measurements of aortic root diameter using the myograph chambers. Aortic root diameters are increased in both male and female MFS mice compared to age- and sex-matched healthy CTRL mice. *Cav1* gene deletion has no impact on artic root growth in both male and female MFS mice aorta. (Means ± SE, N = 6–9 mic/group, Two-Way ANOVA followed by Tukey’s pairwise comparison, *p* ≤ 0.05).

To determine the impact of *Cav1* deletion on aortic root growth and aneurysm progression in male and female MFS mice, we measured the internal diameters of aortic rings isolated from mice. As expected, aortic root diameters are significantly increased in both male and female MFS mice compared to age-matched healthy CTRL mine, with no sex differences observed (Figure 2). Interestingly, *Cav1* deletion had no effects on aortic root growth and aneurysm formation in either male or female MFS/*Cav1KO* groups (Figure 2).

### 2.3. Cav1 Deletion Increases Endothelium-Dependent Vasorelaxation in MFS Mice

**Figure 3:**
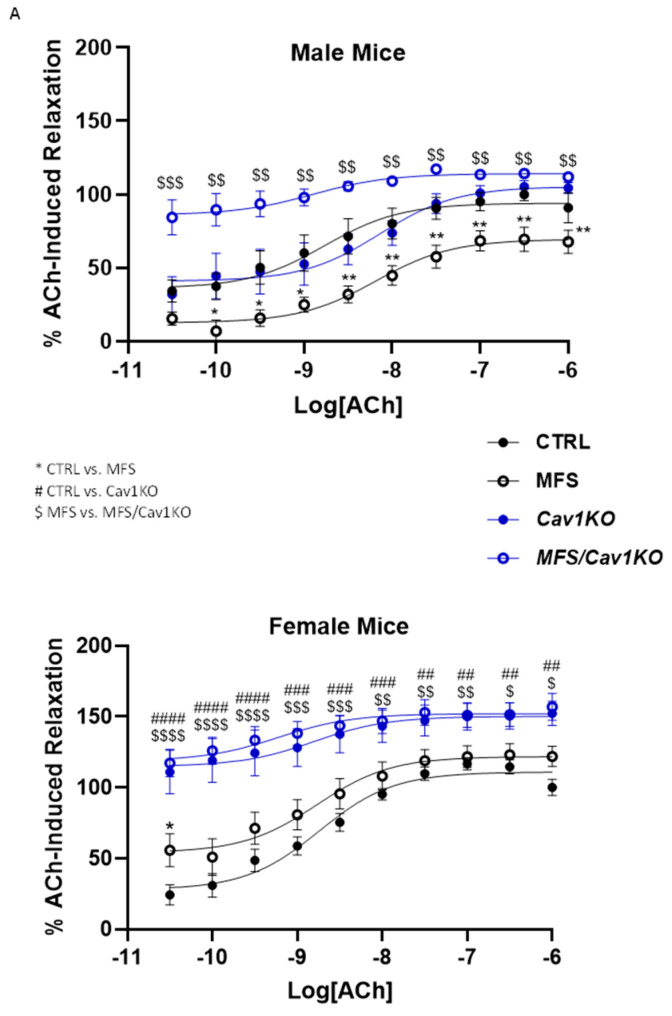

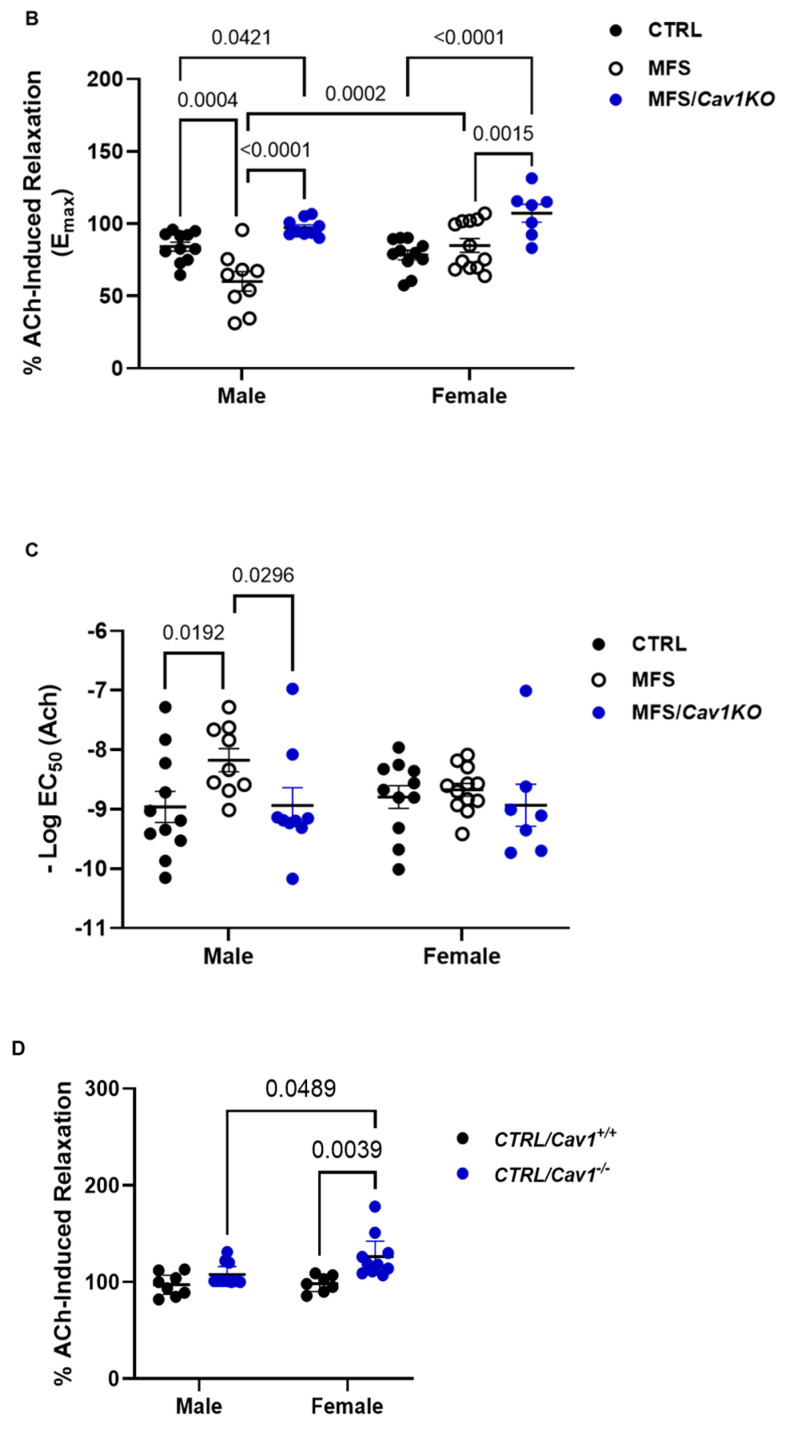
*Cav1* deletion increases endothelium-dependent vasorelaxation in MFS mice. Measurements of acetylcholine (Ach)-induced relaxation in aortic rings isolated from different experimental groups using isometric small chamber myography. (**A**) Dose–response curves (50 pM–1 µM) for acetylcholine (Ach)-induced relaxation in aortic rings isolated from 9-month-old male and female CTRL, MFS, CTRL/*Cav1*KO, and MFS/*Cav1*KO mice. Aortic rings were pre-contracted with sub-max of PE prior to cumulative application of Ach. Relaxation responses were compared to values for CTRL aortic rings that were arbitrarily set as 100% of relaxation. All values were normalized to peak values for PE-induced contraction for each aortic ring (* CTRL vs. MFS, # CTRL vs. CTRL/*Cav1*KO, and $ MFS vs. MFS/Cav1KO, * *p* < 0.05, ** *p* < 0.001, $ *p* < 0.05, $$ *p* < 0.001, $$$ *p* < 0.0001, $$$$ *p* < 0.00001, ## *p* < 0.001, ### *p* < 0.0001, #### *p* < 0.00001). (**B**) The Emax values for Ach were derived from the dose–response curves. (N = 9–12 mice/group). Aortic relaxation in response to Ach is significantly decreased in aortic rings isolated from male MFS mice compared to age- and sex-matched healthy CTRL aorta. Female MFS aortic rings show a higher peak relaxation compared to age-matched male MFS mice. *Cav1* gene deletion leads to an increase in maximum Ach-induced aortic vasorelaxation (Emax) in male and female MFS/*Cav1*KO mice compared to age- and sex-matched MFS groups, indicating significant increases in aortic wall NO production in the absence of Cav1 protein. (**C**) EC50 values for Ach are markedly increased in male MFS mice compared to sex- and age-matched CTRL, but no difference is observed in age-matched female CTRL and MFS aorta. Deletion of *Cav1* results in a significant drop in Ach EC_50_ values in age-matched male MFS/*Cav1*KO aorta compared to MFS mice, indicating an increased sensitivity of aortic endothelial layer to Ach treatment in male mice aortic rings. However, in female mice, *Cav1* deletion does not impact aortic endothelial cell sensitivity to Ach. (**D**) Deletion of *Cav1* increased Ach-induced vasorelaxation in female CTRL mice aorta, with no changes observed in male CTRL subjects. In this bar graph, the average values for Ach-induced vasorelaxation in male CTRL aorta are arbitrarily set as 100% of relaxation. (Means ± SE, N = 9–12 mic/group, Two-Way ANOVA followed by Tukey’s pairwise comparison, *p* ≤ 0.05).

Given the well-documented inhibitory effect of Cav1 protein on endothelial NO production, we assessed the impact of *Cav1* deletion on aortic vasorelaxation in male and female CTRL and MFS mice. In myograph chambers, aortic segments isolated from male and female mice were pre-constricted with the sub-maximum concentration of PE (1 µM), followed by the application of the vasorelaxant agent ACh in a concentration-dependent manner (Figure 3A). As shown in Figure 3A, the initial relaxation response to ACh were significantly higher in both *Cav1KO* and MFS/*Cav1KO* mice compared to CTRL and MFS mice aorta, indicating a much higher level of agonist-stimulated NO production in the aortic rings isolated from mice lacking *Cav1* expression. When comparing the values for the maximal response to ACh-induced vasorelaxation (E_max_), we detected a significant decrease in male MFS mice aortic rings (Figure 3B), with no difference detected between age-matched female CTRL and MFS mice (Figure 3B). It is noteworthy that the E_max_ values for Ach responses were significantly higher in female MFS mice compared to age-matched male MFS mice (Figure 3B). The deletion of *Cav1* resulted in a significant increase in the E_max_ values of ACh in both male and female MFS/*Cav1KO* mice compared to age- and sex-matched MFS groups, indicating that *Cav1* deletion leads to an increase in endothelium-dependent vasorelaxation in MFS mice aortic rings (Figure 3B).

To investigate whether the observed increases in ACh-induced vasorelaxation in male and female MFS/*Cav1KO* mice aorta were due to changes in aortic wall sensitivity to ACh, we calculated the EC_50_ (−Log EC_50_) values for ACh from the dose–response curve (Figure 3A). These data show that the EC_50_ values for the ACh responses in male MFS aorta are higher compared to male CTRL mice, with no differences observed between female CTRL and MFS aortic rings (Figure 3C). The deletion of *Cav1* enhanced the sensitivity of male MFS/*Cav1KO* aorta to ACh-induced vasorelaxation in male MFS/*Cav1KO* mice, with no effects observed in female MFS/*Cav1KO* mice (Figure 3C). Interestingly, in healthy CTRL mice, while *Cav1* deletion caused an increase in aortic relaxation in female CTRL/*Cav1KO* mice, such effects were not detected in male CTRL/*Cav1KO* subjects, suggesting a sex-dependent effect for *Cav1* deletion in CTRL aorta (Figure 3D).

### 2.4. Cav1 Deletion Reduces Phenylephrine-Induced Aortic Contraction in CTRL and MFS Mice

**Figure 4:**
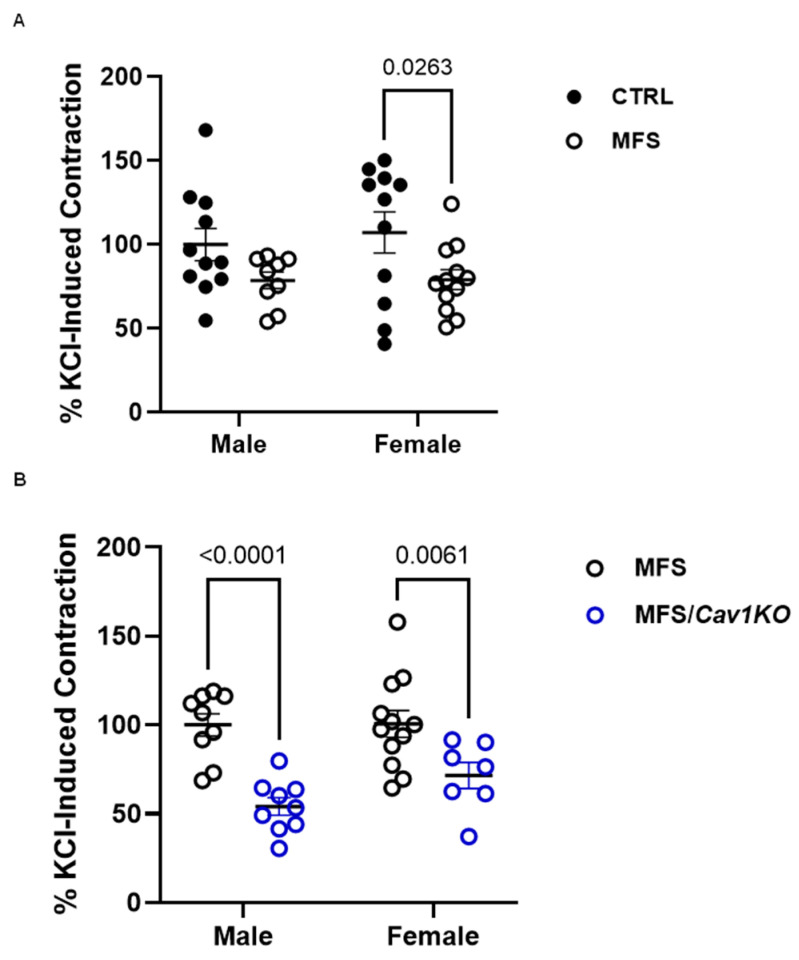
*Cav1* deletion reduces KCl-induced aortic contraction in MFS mice. Bar graphs present maximum force generation in isolated aortic rings in response to KCl (60 mM) in the myograph chambers. (**A**) KCl-induced aortic contraction is reduced in female MFS mice compared to age- and sex-matched controls. No genotype-associated differences are observed in age-matched male CTRL and MFS. The maximum force generated in response to KCl in male CTRL aortic rings was arbitrarily set as 100% of generated force in aortic rings. (**B**) *Cav1* deletion reduces KCl-induced smooth muscle contraction in male and female MFS/Cav1KO mice compared to age- and sex-matched MFS aorta. (Means ± SE, N = 9–12 mic/group, Two-Way ANOVA followed by Tukey’s pairwise comparison, *p* ≤ 0.05).

To ensure aortic ring viability and the smooth muscle contractility of aortic rings, we first evaluated the impact of *Cav1* deletion on smooth muscle membrane depolarization and force generation in a myograph chamber by subjecting isolated aortic rings from 9-month-old male and female CTRL, MFS, and MFS/*Cav1*KO mice to 70 mM KCl (high K^+^ concentration) of the buffer solution. In male mice, high K^+^-induced aortic contraction in response to high K^+^ buffer was not significantly different between CTRL and MFS groups (Figure 4A). On the other hand, in female MFS mice, aortic contraction in response to high K^+^ buffer was significantly lower compared to CTRL groups, highlighting a sex-dependent effect (Figure 4A). The deletion of the *Cav1* gene significantly reduced KCl-induced aortic contraction in both male and female MFS/*Cav1*KO groups, suggesting that Cav1 can potentially regulate calcium entry in aortic smooth muscles independent of sex (Figure 4B).

**Figure 5:**
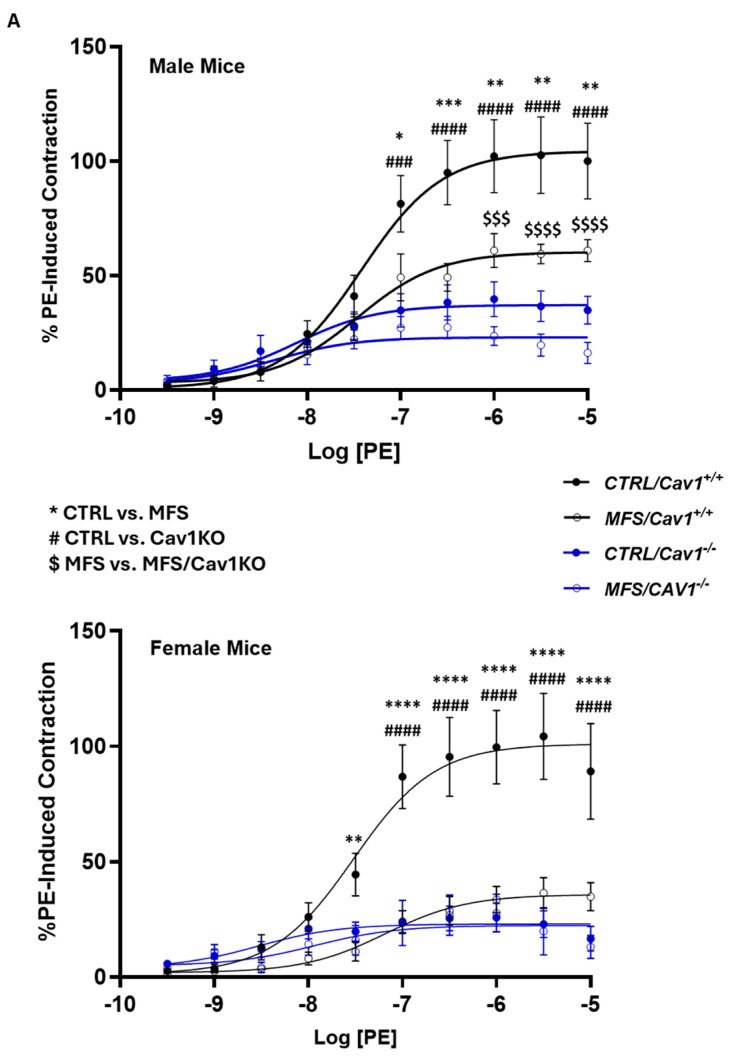

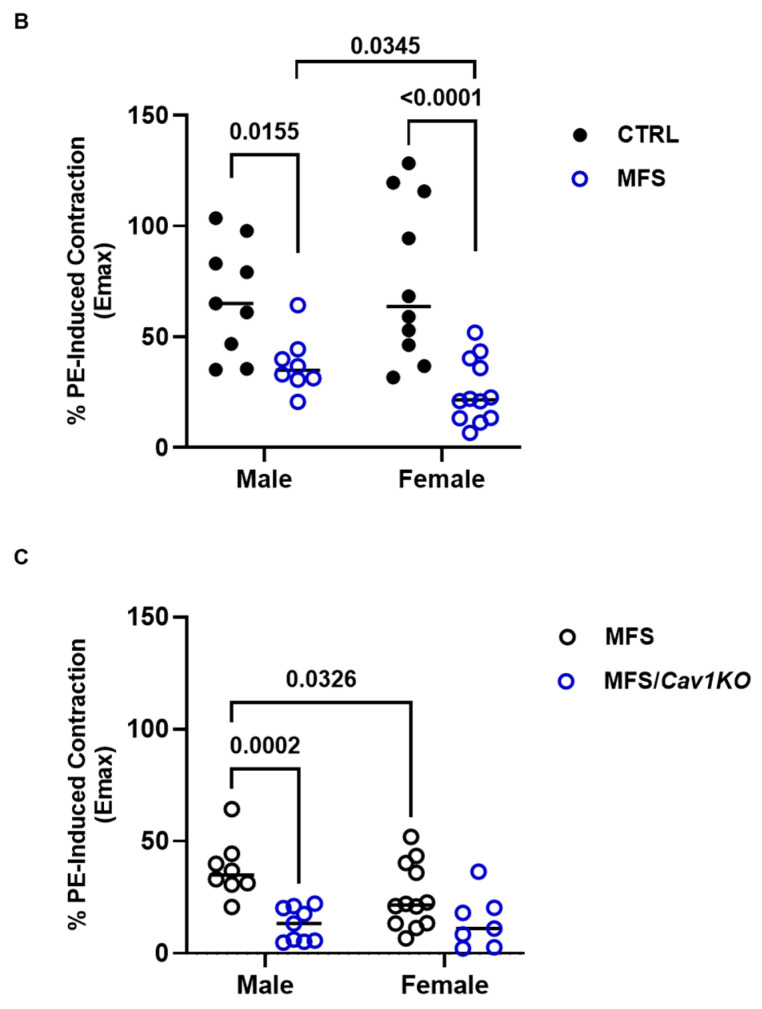
*Cav1* deletion decreases phenylephrine-induced aortic contraction in CTRL and MFS mice. Measurements of mouse aortic ring contractions in response to sub-max concentration (1 µM) of phenylephrine (PE) in the myograph chambers. (**A**) Dose–response curves (1 nM–50 µM) for PE-induced contraction in aortic rings isolated from 9-month-old male and female CTRL, MFS, CTRL/*Cav1*KO, and MFS/*Cav1*KO mice. All values were normalized to peak values for KCl-induced contraction for each aortic ring. Recorded contraction responses were compared to values for CTRL aortic rings that were arbitrarily set as 100% of generated contractile force. (* CTRL vs. MFS, # CTRL vs. CTRL/*Cav1*KO, and $ MFS vs. MFS/Cav1KO, * *p* < 0.05, ** *p* < 0.001, *** *p* < 0.0001, **** *p* < 0.00001, $$$ *p* < 0.0001, $$$$ *p* < 0.00001, ### *p* < 0.0001, #### *p* < 0.00001).). (**B**) In both male and female MFS mice, aortic ring contractions in response to PE are significantly lower compared to healthy CTRL aorta. When we compared age-matched male and female MFS aorta, we observed a significant reduction in female MFS aortic contraction compared to male MFS mice. (**C**) *Cav1* deletion reduces PE-induced aortic contraction in male MFS mice, but not in females, indicating a sex-specific effect of *Cav1* deletion on aortic wall contraction. However, it is important to notice that *Cav1* deletion reduces PE-induced contraction in male MFS aorta to levels that are similar to the values observed in female MFS/Cav1KO mice. (Means ± SE, N = 7–10 mic/group, Two-Way ANOVA followed by Tukey’s pairwise comparison, *p* ≤ 0.05).

To further investigate the impact of *Cav1* deletion on receptor-mediated smooth muscle contraction, we subjected aortic rings isolated from all experimental mice to increasing doses (10 nM–50 µM) of the vasoconstricting agonist PE in myograph chambers, where the maximum contractions achieved in response to PE in CTRL aortic rings were arbitrarily set to 100 percent of contraction (Figure 5A). In both male and female MFS aorta, the maximum PE-induced contractile forces (E_max_) were significantly reduced compared to the age- and sex-matched CTRL subjects (Figure 5B), with female MFS aorta exhibiting lower levels of contraction compared to male MFS aorta (Figure 5C). The ablation of the *Cav1* gene caused further reductions in PE-induced contraction in male MFS/*Cav1KO* mice compared to the male MFS group, but not in female MFS/*Cav1KO* aorta (Figure 5C), indicating a sex-dependent effect.

### 2.5. Cav1 Deletion Reduces Mouse Aortic Contraction by Increasing NO-Dependent Response

**Figure 6:**
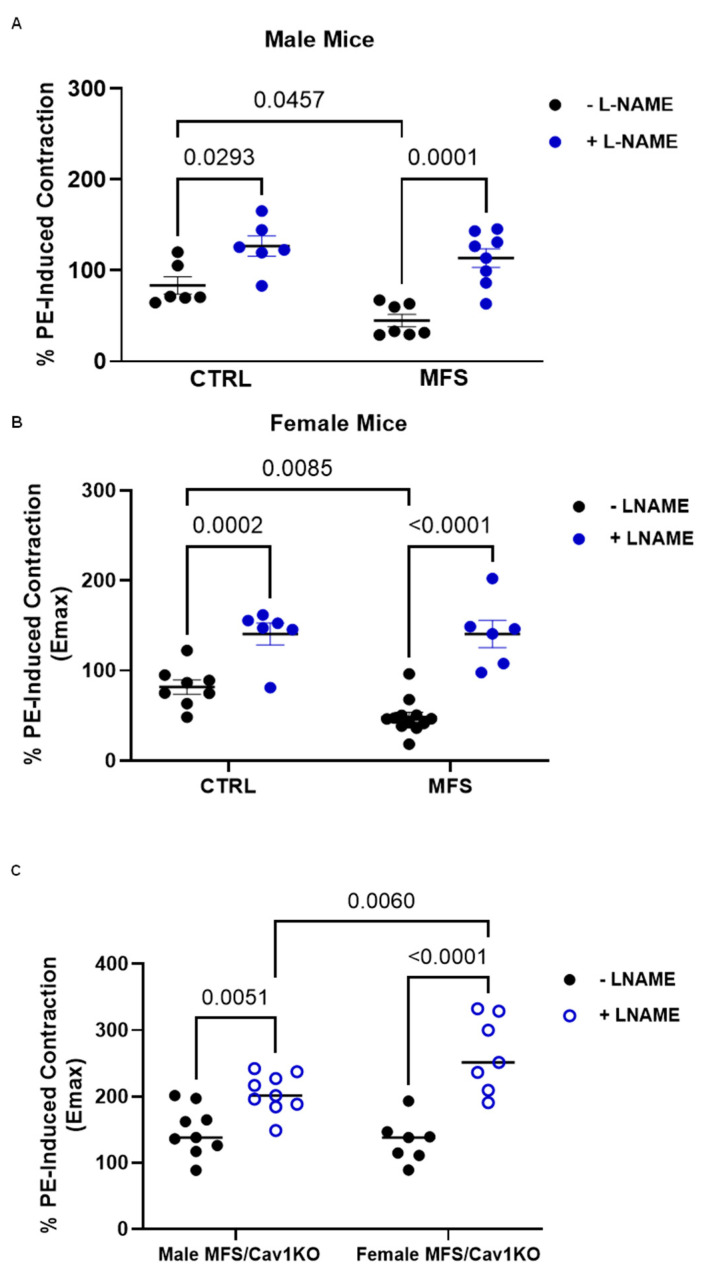
*Cav1* deletion decreases mouse aortic contraction by increasing NO production. Bar graphs present maximum (Emax) aortic contraction in response to sub-maximum concentration (1 µM) of phenylephrine (PE) in CTRL, MFS, and MFS/*Cav1*KO mice in the absence or presence of L-NAME (200 µM), which is a general inhibitor of NO synthesis. (**A**) Pre-treatment of aortic rings with L-NAME increases aortic root contraction in age-matched male CTRL and MFS aorta. (**B**) Pre-treatment of aortic rings with L-NAME also increases aortic root contraction in age-matched female CTRL and MFS aorta. (**C**) L-NAME increases PE-induced aortic contraction in male and female MFS/*Cav1KO* mice. (Means ± SE, N = 6–9 mic/group, Two-Way ANOVA followed by Tukey’s pairwise comparison, *p* ≤ 0.05).

To investigate whether the *Cav1KO*-mediated decrease in aortic contraction is facilitated through an increase in the NO production, aortic segments were pre-incubated with a non-selective NOS inhibitor L-NAME (200 µM) in the myograph chambers for 30 min before the application of PE. The pre-treatment of aortic rings with L-NAME resulted in significant increases in PE-induced vasocontraction peaks in both male (Figure 6A) and female (Figure 6B) CTRL and MFS mice aorta, likely due to an L-NAME-mediated decrease in the endogenous NO within the aortic wall. Furthermore, in both male and female MFS/*Cav1KO* mice, the pre-treatment of aortic rings with L-NAME enhanced contractile responses to PE (Figure 6C) compared to MFS mice, indicating that *Cav1* deletion caused an even more significant increase in basal NO levels in MFS mouse aortic rings, further highlighting the notion that Cav1 regulates MFS mouse aortic contraction through NO-dependent mechanisms. Our data also showed that L-NAME caused much higher increases in aortic rings isolated from female MFS/*Cav1KO* mice compared to male subjects (Figure 6C). This confirms higher levels of NO-dependent response during smooth muscle contraction in female MFS aortic rings.

### 2.6. Cav1 Deletion-Mediated Increase in Aortic NO-Dependent Response Is Not Through Inducible Nitric Oxide Synthase (iNOS)

**Figure 7:**
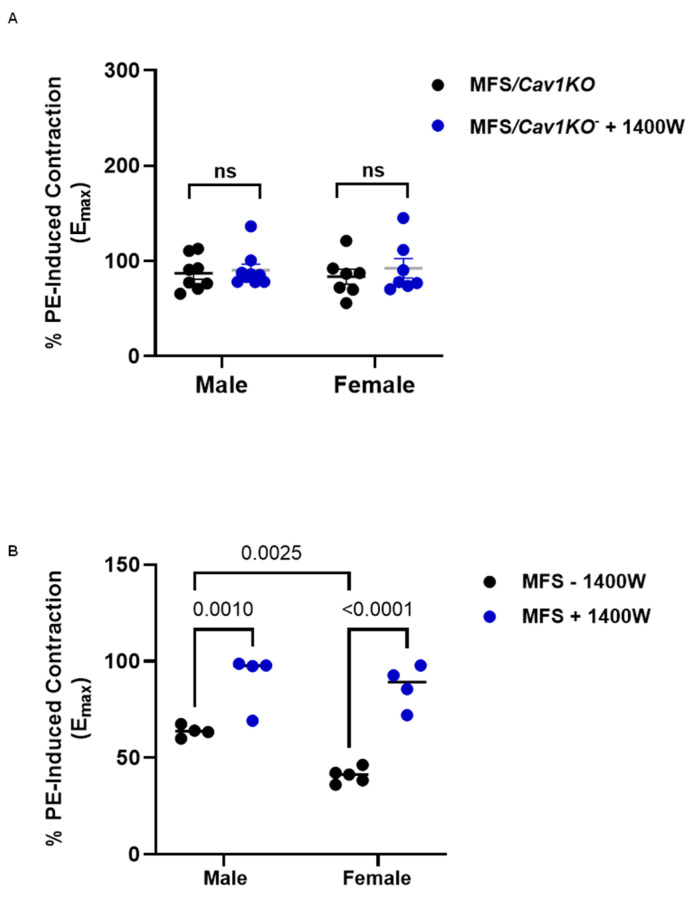
*Cav1* deletion-mediated increases in aortic NO production are not through iNOS. Presented bar graphs showcase the impact of 1400 W, a potent inhibitor of inducible NOS (iNOS) on maximum (Emax) PE-induced contraction in aortic rings isolated from male and female MFS/*Cav1*KO mice. (**A**) Pre-treatment of MFS/*Cav1*KO mice aortic rings with the iNOS inhibitor 1400 W (1 µM) has no effects on PE-induced aortic contraction in age-matched male and female MFS/*Cav1*KO mice, indicating that the excessive increase in aortic endogenous NO production in the absence of Cav1 is not mediated by iNOS activation. (**B**) Pre-treatment of CTRL and MFS mice aortic rings with the iNOS inhibitor 1400 W (1 µM) significantly increased PE-induced vasoconstriction in both male and female MFS mice aorta, indicating that iNOS-mediated NO response elevated in male and female MFS aorta. (Means ± SE, N = 6–8 mic/group, Two-Way ANOVA followed by Tukey’s pairwise comparison, *p* ≤ 0.05).

To identify the source for increased basal and endogenous NO in our MFS/*Cav1KO* model, we utilized a potent and specific inhibitor of iNOS, N-3-Aminomethyl benzyl acetamidine (1400 W), in the myograph chamber. In our experiments, aortic rings were pre-incubated with 1400 W (1 µM) for 30 min prior to PE application. The pre-treatment of aortic rings with the iNOS inhibitor did not impact the *Cav1*KO-mediated decrease in aortic contraction in either male or female MFS/*Cav1*KO aortic rings (Figure 7A). This important observation implies that iNOS expression is not affected by the *Cav1* gene deletion in MFS mice. Interestingly, the pre-treatment of MFS mouse aortic rings with 1400 W increased PE-induced contraction (Figure 7B). This observation is in line with previous reports of iNOS increases in MFS aortic tissue [43,44,45,46].

### 2.7. Cav1 Deletion Improves Aortic Wall Strength and Elastin Structure in Male MFS/Cav1KO Mice

**Figure 8:**
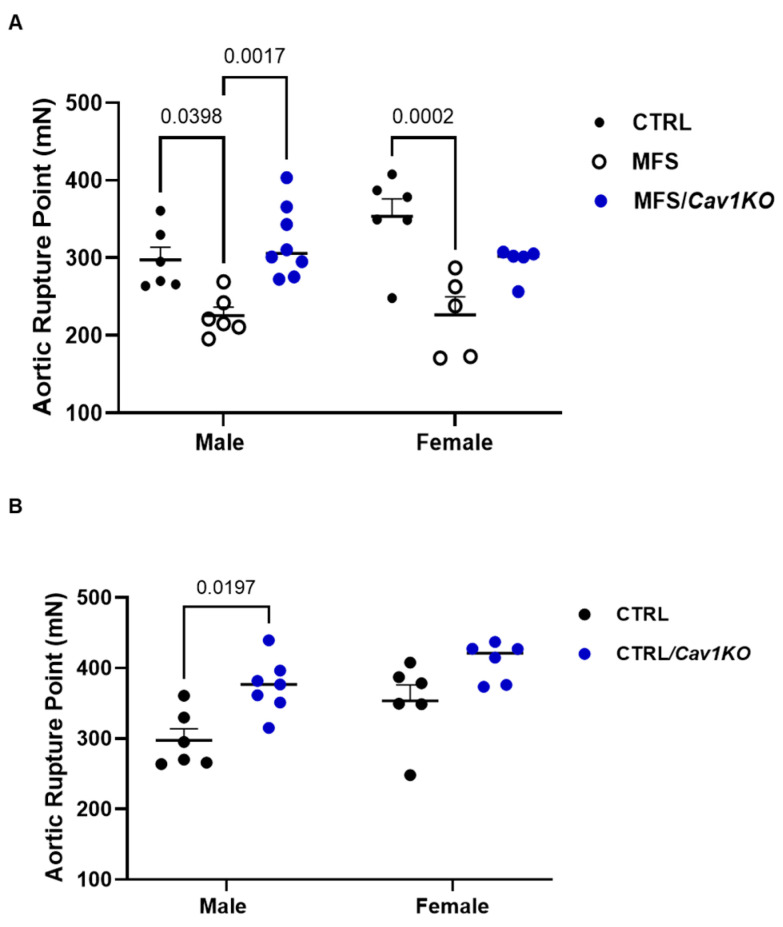
*Cav1* deletion markedly improves aortic wall strength in male MFS/Cav1KO mice. Scatter plot graphs show the force generated (mN) at the rupture point. The rupture point of these aortic segments represents the maximum force generated by each segment at the point of maximum stretch, just before the aortic wall ruptures. (**A**) As expected, aortic wall rupture point is significantly reduced in both male and female MFS groups compared to age- and sex-matched healthy CTRL mice. Deletion of *Cav1* gene increases the aortic ring rupture points in male CTRL mice, with no effects observed in female CTRL subjects. (**B**) Similarly, *Cav1* deletion further increases aortic wall strength (rupture point) only in male MFS mice, indicating a sex-dependent effect. (Means ± SE, N = 4 mic/group, Two-Way ANOVA followed by Tukey’s pairwise comparison, *p* ≤ 0.05).

It is well established that MFS aneurysm is associated with aortic wall weakening and the fragmentation of elastin fibers within the aortic wall. To determine the effects of the *Cav1* gene deletion on aortic wall strength, the aortic segment rupture points were assessed in all experimental groups using small vessel isometric myography. The rupture point of aortic segments represents the maximum force generated by each segment at the point of maximum stretch, just before the aortic wall ruptures. Based on our findings, both male and female MFS mice demonstrated decreased aortic wall strength (as evidenced by reduced aortic wall force generation at the rupture point) compared to the age- and sex-matched CTRL mice (Figure 8A). When MFS aortic rings are compared with MFS/*Cav1KO* aorta, only male MFS/*Cav1KO* mice showed improvements in the maximum aortic wall strength (Figure 8A). Similarly, when CTRL mice aorta was compared with CTRL/*Cav1KO* groups, only male, but not female CTRL/*Cav1KO* mice show an improvement in the maximum wall strength (Figure 8B), suggesting that the effects of *Cav1* deletion on aortic wall strength and structural integrity in both CTRL and MFS mice are sex dependent.

**Figure 9:**
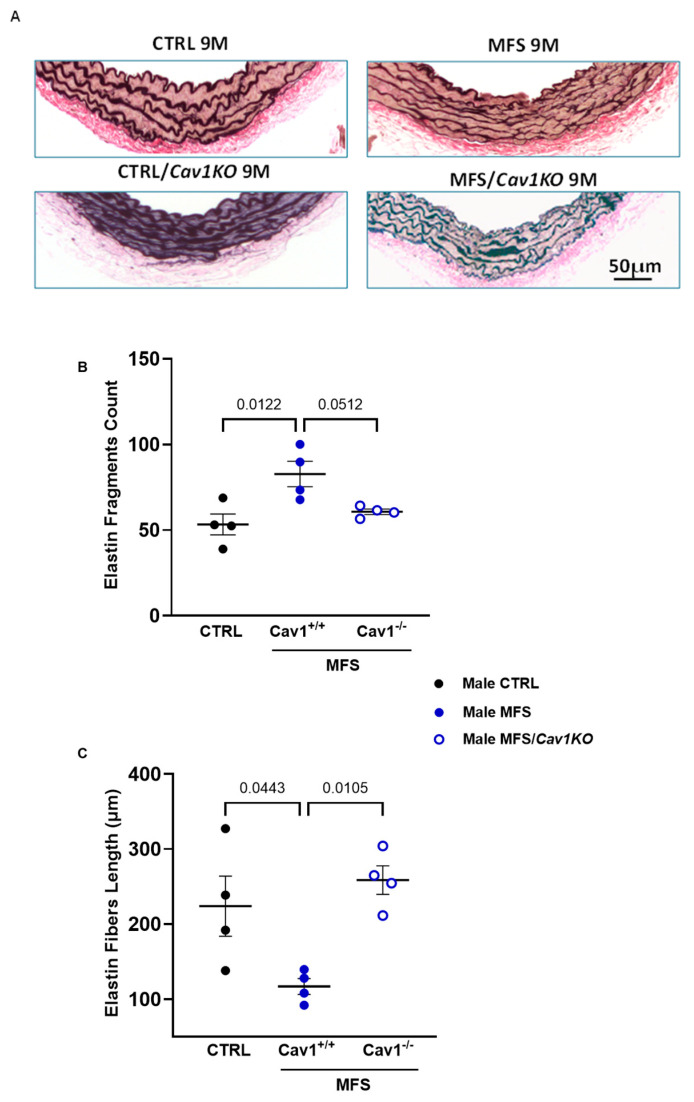
*Cav1* deletion improves aortic wall elastin structure in male MFS/*Cav1*KO mice. Data presents measurements of aortic wall elastin fragment counts and length within the aortic wall sections isolated from CTRL, MFS, and MFS/*Cav1*KO mice. (**A**) Representative images showing Van Gieson staining of 10 µm aortic sections isolated from male CTRL, MFS, CTRL/*Cav1*KO, and MFS/*Cav1*KO mice, showing elastin in dark purple in the medial layer of mouse aortic wall (scale bar = 50 µm). (**B**) Quantification of elastin fiber counts shows a significant decrease in fragment counts in male MFS/*Cav1*KO aorta compared to MFS mice. (**C**) Similarly, *Cav1* deletion reduces elastin fiber fragment length in MFS mice, indicating a marked improvement in elastin structure within the aortic wall of MFS/*Cav1*KO mice. (Means ± SE, N = 4 mic/group, Two-Way ANOVA followed by Tukey’s pairwise comparison, *p* ≤ 0.05.

In MFS, an aortic root aneurysm is associated with aortic wall medial elastin fragmentation. Since the *Cav1* deletion only improved aortic wall strength in male mice, we performed a further investigation to determine the effects of the *Cav1* deletion on aortic wall elastin structural integrity in male MFS mice using van Gieson staining, where elastin fibers were stained in dark purple in the medial layer of the aortic wall (Figure 9A). As expected, male MFS mice showed increased elastin fragmentation (Figure 9B) and decreased elastin fiber length (Figure 9C) in the aortic wall compared to male CTRL mice. Notably, *Cav1* deletion significantly improved the aortic elastin structure in male MFS/*Cav1KO* mice. This was demonstrated by a reduction in the number of elastin fragments (Figure 9B) and an increase in the length of elastin fibers (Figure 9C) within the aortic wall, suggesting improved elasticity in the aortic wall of male MFS/*Cav1KO* mice.

## 3. Discussion

The present study aimed to investigate the potential sex-specific role of Cav1 protein in regulating physiological responses pertinent to the progression of MFS-associated aortic root growth, aortic wall structural integrity, and aortic endothelial and smooth muscle function. Cav1 is a critical scaffolding protein predominantly found in the plasma membrane’s caveolae, which are small, flask-shaped invaginations rich in cholesterol and sphingolipids [47,48]. Cav1 is integral to various cellular processes, including signal transduction, lipid regulation, inflammatory responses, and vesicular transportation [29,49], while directly interacting with a wide range of signaling proteins to modulate their activity [50]. In addition to its role in normal cellular function, alterations in Cav1 expression and function are implicated in several pathophysiological conditions, such as inflammation [51], cancer [52], cardiovascular diseases [53,54], and diabetes [55,56]. In the vasculature, Cav1 interacts with cell membrane’s signaling molecules such as G-protein coupled receptors, insulin receptors, and cholesterol via its Cav scaffolding domain (CSD) and most importantly, has critical roles in regulating NO production within the vascular wall [57,58,59]. Notably, Cav1 attenuates eNOS activity within the vascular wall, thereby influencing vascular tone and endothelial function.

In individuals with MFS, the most common cause of mortality and morbidity is aortic root aneurysm, dissection, and rupture. MFS is caused by mutations in the *Fbn1* gene, leading to increased TGF-β signaling, reduced smooth muscle contractility, and impaired endothelial function, all of which contribute to the development of aortic aneurysms [1,8]. Previous studies indicate that Cav1 plays a significant role in regulating both eNOS and TGF-β signaling within the vascular wall and in the development of angiotensin II (AngII)-induced abdominal aortic aneurysms (AAAs) [60,61,62,63]. However, the role of Cav1 in MFS-associated aortic root aneurysms has yet to be investigated.

Our data clearly show that *Cav1* deletion induces a significant increase in endothelium-dependent relaxation in both male and female MFS mice, further confirming the inhibitory effects of Cav1 on endogenous endothelial NO production (Figure 3A,B). The EC_50_ values for ACh in aortic rings show that the increase in vasorelaxation in male MFS/*Cav1KO* aorta is, at least in part, due to the increased sensitivity of aortic endothelial cells to the vasorelaxant agent (Figure 3C). In line with this observation, we also found further marked decreases in PE-induced vasoconstriction in male MFS/*Cav1KO* mice aorta that were NO-dependent (Figure 5C). Interestingly, in female MFS mice, *Cav1* deletion did not impact PE-induced contraction (Figure 5C). This may be explained by the observation that in female MFS mice, aortic contraction is already significantly lower compared to male MFS mice (Figure 5B), reaching a threshold so low that is not further affected by the deletion of the *Cav1* gene. In both male and female CTRL and MFS aortic rings, pre-treatment with L-NAME increased aortic contraction. This phenomenon is expected as L-NAME inhibits NO-dependent vasorelaxation within the aortic wall. The most intriguing observation is that the application of L-NAME caused a greater potentiation of the PE-induced vasoconstriction in both male and female MFS/*Cav1KO* mice, with a larger response observed in female subjects (Figure 6C). The effect of the nonselective NOS inhibitor L-NAME on PE-induced contraction in both male and female MFS/*Cav1KO* mice further supports the notion that in the absence of the *Cav1* gene expression, basal NO levels and NO-dependent responses are significantly elevated in aortic tissue, leading to a marked reduction in aortic contraction.

The interaction between Cav1 and eNOS is a critical factor in regulating vascular function. In healthy blood vessels, Cav1 attenuates eNOS, reducing the production of NO, maintaining the basal vascular tone, and preventing excessive vasodilation [49]. Under pathological conditions, the interplay between Cav1 and eNOS can be a key factor in disease progression. For example, in conditions such as hypertension or type 2 diabetes, elevated oxidative stress and inflammation can lead to increases in Cav1 protein expression, resulting in a significant decrease in NO bioavailability and production, thereby contributing to endothelial dysfunction, impaired vascular tone, and increased vascular resistance [56,64].

In our study, the specific iNOS inhibitor 1400 W had no impact on MFS/*Cav1KO* mouse aortic contraction (Figure 7), leading to our conclusion that eNOS and/or neuronal NOS (nNOS) are the most plausible sources for increased NO production within the aortic wall. Of these two potential candidates, several lines of evidence support the argument that eNOS is probably the primary target of *Cav1* deletion in the MFS mouse model. It has been consistently shown that eNOS is predominantly expressed in endothelial cells, playing crucial roles in normal endothelial function and vascular tone [65]. In addition, the deletion of *Cav1* is known to disrupt its inhibitory interaction with eNOS, leading to increased eNOS activity and NO production [22,51].

On the other hand, nNOS is primarily expressed in nerve terminals, skeletal muscle, and, to a lesser extent, in vascular SMCs, and its contribution to systemic vascular NO production is typically less significant compared to eNOS [66]. Our findings also align with previous studies that have demonstrated enhanced eNOS activity in the absence of Cav1, reinforcing the hypothesis that eNOS is the main source of the excessive NO-dependent response observed in MFS/*Cav1KO* mice [67,68]. Further investigations using a more specific and targeted genetic manipulation of the *Cav1* gene would elucidate the mechanisms in the regulatory action of Cav1 on eNOS in the mouse model of MFS-associated aortic aneurysms.

In MFS mice, the progression of aortic aneurysms has been linked to dysregulated NO signaling, involving both eNOS and iNOS. Recent studies have shown that MFS aneurysms are associated with reduced eNOS activity, impairing protective NO signaling, while iNOS activity is elevated, potentially contributing to inflammation and vascular damage [69]. Our data suggest that the knockout of *Cav1* enhances the NO-mediated response by alleviating the Cav1-mediated inhibition of eNOS. However, the increased iNOS activity in MFS may persist independently, contributing to aneurysm progression [70]. However, the elevated iNOS activity in MFS may persist independently, driving aneurysm progression. Therefore, Cav1 may have a dual regulatory role, enhancing NO production by eNOS in its absence, but failing to mitigate the detrimental effects of increased iNOS-derived NO. This complex interaction could be a critical factor in the pathophysiology of MFS aneurysm progression. Given the complex interplay between eNOS and iNOS, along with the potential for eNOS uncoupling in the MFS aorta, and our findings that the iNOS inhibitor 1400 W had no observed effect on aortic contraction in MFS/*Cav1KO* mice, further investigation is critical to elucidate these mechanisms. Such research will be key to understanding how these NOS isoforms interact and influence vascular function in the context of MFS and Cav1 deficiency.

In MFS-associated aortic root aneurysms, SMCs undergo a phenotypic shift marked by the reduced expression of contractile proteins and the increased expression of matrix-remodeling genes, as observed in MFS-modulated cell clusters enriched for extracellular matrix, unique to aneurysmal MFS tissue [71]. These cellular transformations highlight complex interactions between structural and signaling changes, which not only weaken the aortic wall but may also offer potential targets for therapeutic intervention in MFS aneurysms.

Interestingly, Cav1 influences the SMC phenotype by maintaining a contractile state, as Cav1 loss is associated with SMC de-differentiation and a shift toward a synthetic phenotype [33], which is less contractile and more prone to extracellular matrix production—a hallmark of aneurysmal progression in MFS [71]. Furthermore, Cav1 regulates calcium signaling [72] and localizes growth factor receptors within caveolae, thus modulating SMC contractility and the response to growth stimuli [33]. The disruption of these pathways due to Cav1 deficiency contributes to vascular wall weakening, reinforcing Cav1’s essential role in supporting SMC function and vessel integrity. These mechanisms collectively suggest that Cav-1 is pivotal in maintaining SMC contractility.

Although a reduction in aortic wall strength in MFS mice is evident in both sexes, the response to *Cav1* deletion seems to be sex-specific in MFS/*Cav1KO* mice. In our study, we found that *Cav1* deletion enhanced aortic wall strength only in male MFS, as evidenced by the improved aortic wall rupture points (Figure 8). The observed difference could be due to different interactions between female and male sex hormones with pathways regulated by Cav1 protein. It is reported that Cav1 protein co-localizes and interacts with both the NH2-terminal and the ligand-binding domains of the androgen receptor (AR), and that the downregulation of Cav1 can reduce AR activity [73,74,75]. Androgens can also stimulate the synthesis of collagen in the vascular wall. Increased androgen levels have been associated with higher collagen deposition and reduced elastin content, leading to decreased elasticity [76,77]. Interestingly, Doubacher et al. have shown that increased resistance to rupture and improved aortic mechanical properties in the transgenic mouse model of MFS can be attributed to collagen content, but not elastin [78]. These reports may explain the observed protective effects of *Cav1* deletion on aortic wall strength in male MFS mice.

The sex-specific action of *Cav1* deletion may also be explained by its interaction with estrogen receptor alpha (ERα). Cav1 may physically associate with ERα within caveolae, leading to post-translational modifications that are necessary for the initiation of estrogen signaling pathways and its downstream beneficial effects on vascular function [79,80]. In females, estrogen can modulate the expression and activity of various proteins involved in vascular function, including eNOS [81]. It is possible that in female MFS mice, the presence of estrogen might mitigate the effects of *Cav1* deletion on aortic wall strength by differentially regulating pathways related to vascular remodeling and repair. Additionally, genes involved in ECM organization and inflammatory responses to injury (e.g., aortic root aneurysm) might be regulated differently in females than in males, leading to sex-dependent outcomes following *Cav1* deletion. Although we have shown that *Cav1* deletion can increase aortic NO-dependent responses in both male and female MFS mice, the downstream effects of enhanced NO signaling might differ in males and females.

Our findings also suggest that *Cav1* deletion could improve the aortic wall elastin fiber structure within the aortic wall of MFS/*Cav1KO* mice (Figure 9). The structural integrity of elastin fibers is crucial for supporting aortic wall elasticity. Elastin synthesis and structure can be directly or indirectly controlled by multiple signaling pathways originating from the ECM or cell–ECM interactions. In MFS, *Fbn1* mutation can lead to an increase in TGF-β bioavailability and downstream signaling within the aortic wall. Excessive TGF-β signaling results in increased cell proliferation, differentiation, apoptosis, oxidative stress, and elastin degradation, all contributing to the development of multiple MFS manifestations [5,82,83]. The inhibitory effect of Cav1 on both the TGF-β and AT1R downstream signaling pathways can reduce the inflammation resulting from the secondary cascade and the downstream elastin fragmentation and loss of aortic wall elasticity [84]. Hence, we expected that *Cav1* deletion would exacerbate elastin fragmentation in the aortic wall in MFS/*Cav1KO* mice.

Intriguingly, we found that in male MFS/*Cav1KO* mice, *Cav1* deletion caused a significant increase in aortic wall strength (as evidenced by an increase in wall rupture point) and an improvement in aortic wall elasticity (as evidenced by increases in elastin fiber length and reduction in elastin fragments count within the aortic wall), with no beneficial effects observed in female subjects (Figure 8). Our observations contradict previous reports suggesting that *Cav1* knockout would decrease TGF-β and MMP expression, therefore reducing elastin degradation [85,86]. The unexpected and sex-dependent phenomenon in male MFS/*Cav1KO* mice raises new questions about the regulatory impact of Cav1 on the downstream signaling pathways involved in ECM turnover, including TGF-β and MMPs. It is plausible that in MFS male mice, the absence of Cav1 protein alters the regulation of TGF-β signaling, stabilizing the ECM and reducing elastin fragmentation. This hypothesis requires validation through further molecular and pharmacological studies.

**Figure 10:**
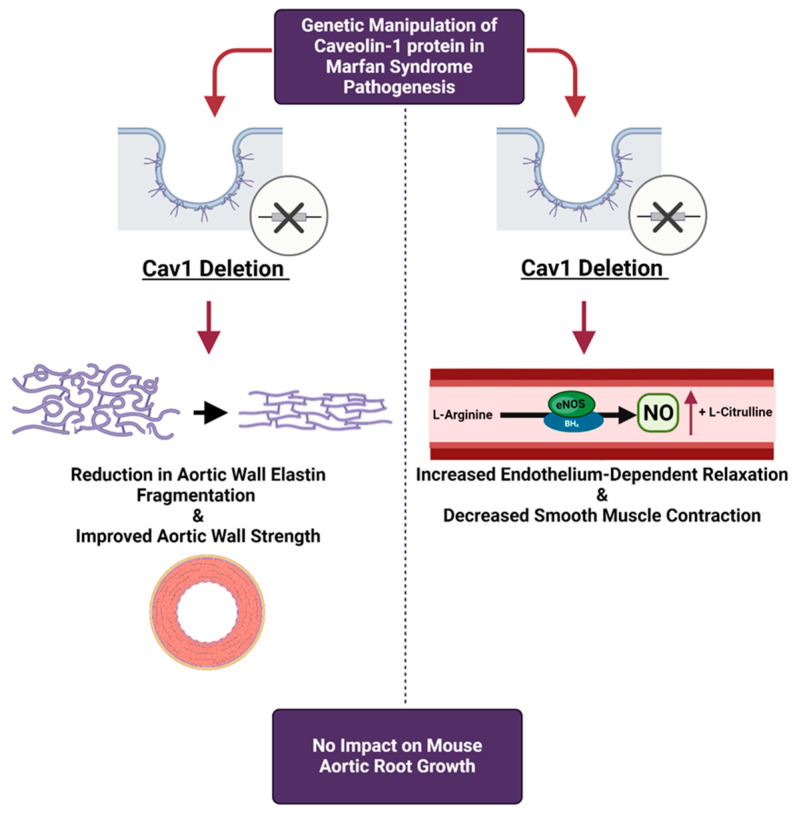
Potential impact of *Cav1* ablation on MFS aeropathy. In MFS mice, *Cav1* deletion results in improvement in aortic wall elastin structure and aortic wall strength. The deletion of *Cav1* also increases endothelium-mediated vasorelaxation in the aorta, while decreasing smooth muscle contraction in response to vasoconstricting agent phenylephrine. The overall conclusion is that *Cav1* deletion provides some levels of beneficial impacts on aortic structure and function in MFS mice. The image was generated by Biorender (Toronto, ON, Canada).

Based on the present data, we can only conclude that *Cav1* deletion provides beneficial effects on endothelial and SMC function in both male and female MFS mice while improving aortic wall strength and elastin structural integrity only in male subjects (Figure 10). It is also important to point out that despite the observed beneficial effects of *Cav1* deletion on endothelial and SMC function, we did not see any beneficial and protective effects on aortic root growth, raising questions about other regulatory mechanisms that contribute to the development of aortic root aneurysms in MFS mice.

### Study Limitations

The current study has several limitations. The use of a mouse model and the specific experimental approach do not account for potential compensatory mechanisms by other isoforms of Caveolin, such as Cav2 and Cav3. Additionally, the systemic manipulation of *Cav1* gene expression in this study does not target a specific tissue of interest such as the aortic wall endothelium or the smooth muscle layer. A tissue-specific approach would provide a more detailed understanding of the role of Cav1 in specific cell types and their contribution to MFS-associated aortic aneurysms. Future research should also incorporate more specific measurements of TGF-β, MMPs, eNOS, and iNOS expressions in the aortic sections and blood samples of MFS/*Cav1KO* mice. Such targeted studies will help clarify the role of Cav1 in regulating ECM dynamics and aeropathy in MFS. Finally, this study only investigates the effects of *Cav1* deletion on aortic root structure and function in 9-month-old CTRL and MFS mice. A longitudinal study that includes different age groups will further clarify the timeline for the observed protective effects.

## 4. Materials and Methods

### 4.1. Experimental Mouse Model

The MFS mouse model is heterozygous for an *Fbn1* allele (*Fbn1^C1041G/+^*), replicating the phenotype of aortic root aneurysm associated with the most common mutation seen in MFS aneurysm patients [21]. A breeding colony of MFS mice was established in the Midwestern University Animal Facility. The female *Cav1*KO mouse (B6.Cg-*Cav1^tm1Mls^*/J) was purchased (Jackson Laboratory, Bar Harbor, ME, USA) and crossed with male MFS mouse (*Fbn1^C1041G/+^*) through multiple backcrossings to generate MFS mice lacking the *Cav1* gene (*FBN1^−/+^/Cav1^−/−^*) that are referred to MFS/*Cav1KO* throughout the report. In brief, we bred enough numbers of control and MFS mice by crossing female control (*FBN1^+/+^/Cav1^+/+^*) mice with male MFS (*FBN1^−/+^/Cav1^+/+^*) to avoid any pregnancy-related complications that are common in female MFS mice. To produce MFS/*Cav1KO* mice, we first crossed male MFS mice (*FBN1^−/+^/Cav1^+/+^*) with female *Cav1KO* mice (*FBN1^+/+^/Cav1^−/−^*) that resulted in generation of MFS mice heterozygous for *Cav1* gene (*FBN1^−/+^/Cav1^+/−^*). The F2 mice were generated by backcrossing male *FBN1^−/+^/Cav1^+/−^* mice with female *Cav1KO* mice (*FBN1^+/+^/Cav1^−/−^*) that resulted in generating MFS mice lacking *Cav1* gene (*FBN1^−/+^/Cav1^−/−^*). The F3 generation of mice was bred by backcrossing male MFS/*Cav1*KO (*FBN1^−/+^/Cav1^−/−^*) with female *Cav1KO* mice (*FBN1^+/+^/Cav1^−/−^*). A parallel breeding colony was also established by crossbreeding male MFS (*Fbn1^C1041G/+^*) and female wildtype (*C57BL/6J*) mice to produce equal numbers of MFS and wildtype mice for the study.

To determine and confirm mouse genotype, mouse tail samples were sent to Transnetyx (Transnetyx, Cordova, TN, USA) for verification by PCR. Following genotyping, mice were divided into experimental groups (N = 7–10) as follows: male and female wildtype control (CTRL), MFS, *Cav1* KO, and MFS/*Cav1* KO. Mice (4 per cage) were housed under standard animal room conditions (a 12 h light and dark cycle, 25 °C) and fed on a standard Teklad Global 18% protein rodent diet (Envigo (Inotiv), Indianapolis, IN, USA). This study was conducted in strict accordance with international guidelines and standards for the care and use of animals in research, ensuring that the highest ethical considerations were met throughout the experimental process. All animal care, breeding, and surgical procedures were also performed according to guidelines set by the National Institute of Health and the Midwestern University Institutional Animal Care and Use Committee [approved IACUC protocols MWU-2635 and MWU-2636].

### 4.2. Sample Size Calculations

Sample size calculations were performed for different sets of experiments. For isometric myography experiments, the calculation was based on detecting a 20% decrease in aortic root diameter (lumen size) that corresponds to the threshold for surgical intervention in MFS aneurysm patients. The same parameters were used in our previous published reports using the same transgenic animal model of MFS. Based on our previous data, we performed a power analysis using G*Power 3.1, with an alpha level of 0.05 and a power of 0.90, resulting in a required sample size of minimum of 7–8 mice per group. For histological staining of elastin, we performed the calculation based on detecting a 20% decrease in elastin fragment counts within the aortic wall using our previously published data. The G*Power calculation, with an alpha level of 0.05 and a power of 0.90, resulted in a required sample size of minimum of 4 mice per group.

### 4.3. Aortic Tissue Preparation

At 9 months of age, mice from all experimental groups were euthanized with 5% isoflurane and 2 L/min of 100% oxygen inhalant followed by cervical dislocation. The thoracic cage was collected and placed in ice-cold aerated (95% O_2_ + 5% CO_2_) HEPES-PSS. Later, the whole length of thoracic aorta was dissected, cleaned of fat, and cut into 2 mm segments (rings), with special care to protect the vessel’s endothelial layer. The ascending aorta (from root to arch) was also collected and fixed in 10% formalin buffer for histological processing and staining.

### 4.4. Buffers and Reagents

Aortic segments were dissected, cleaned, and incubated in HEPES Physiological Salt Solution (HEPES-PSS) buffer (pH 7.43) containing 10 mM HEPES, 6 mM glucose, 1.8 mM CaCl_2_, 130 mM NaCl, 4 mM KCl, 4 mM NaHCO_3_ 1.2 mM MgSO_4_, 1.18 mM KH_2_PO_4_, and 0.03 mM EDTA to simulate physiological conditions. The high KCl (K^+^) buffer (pH 7.43) contained 65 mM NaCl, 80 mM KCl, 1 mM MgCl_2_, 10 mM glucose, 5 mM HEPES, and 1.5 mM CaCl_2_ and was used to assess smooth muscle viability and contractile ability. All pharmacological agonists and blockers, including vasoconstricting agent phenylephrine (PE), vasodilatory agent acetylcholine (ACh), non-specific and reversible non-selective NOS inhibitor L-N^G^-Nitro arginine methyl ester (L-NAME), and specific and potent inhibitor of iNOS, 1400 W dihydrochloride, were purchased from Sigma Millipore (Sigma Millipore, St. Louis, MO, USA). All chemicals were dissolved in HEPES-PSS buffer.

### 4.5. Transmission Electron Microscopy

We used transmission electron microscopy (TEM) to visualize caveolae structure in the mouse aortic wall. Aortic tissues were harvested from experimental mice, fixed in 2.5% glutaraldehyde in 0.1 M phosphate buffer (pH 7.4) for 24 h at 4 °C, and post-fixed in 1% osmium tetroxide. Tissues were then dehydrated through a graded ethanol series and embedded in epoxy resin. Ultrathin sections (70–90 nm) were cut using an ultramicrotome, collected on copper grids, and stained with uranyl acetate and lead citrate. TEM images were captured using a Philips CM 12 electron microscope (Semion LLC, Hillsboro, OR, USA) operating at 80 kV.

### 4.6. Measurements of Aortic Contraction and Vasorelaxation

From each experimental mouse, four aortic segments (2 mm in length) were mounted to a 4-unit chamber myograph for ex vivo study (DMT-USA Inc., Ann Arbor, MI, USA). Force readings were generated and recorded via LabChart 7.0 software (ADinstruments, Colorado Springs, CO, USA). Segments were rested for 30 min in continuously aerated 37 °C HEPES-PSS (gassed continuously with 95% O_2_ and 5% CO_2_) and then stretched to their optimal tension of 6 mN, as established by previous studies for C57BL/6 wildtype and MFS mice [87]. Aortic segment viability was determined by challenging with high potassium chloride (KCl, 60 mM) buffer twice, with 15 min of rest between each challenge. To assess aortic smooth muscle contractile force, segments were challenged with increasing concentrations (1 nM–50 µM) of vasoconstricting agent phenylephrine (PE). For data analysis, the peak values for PE-induced vasoconstriction were normalized over the peak values obtained from high potassium buffer-induced contractions. By normalizing PE-induced contraction against a common standard (high potassium buffer) that elicits a maximal depolarization and contraction response, we ensured that the observed differences in PE-induced contraction are not merely a result of inherent differences in the contractile potential of the tissue, but rather a true reflection of the specific response to PE stimulation. To assess endothelial function and endothelium-mediated vasorelaxation, pre-constricted segments were subjected to increasing concentrations (50 pM–1 µM) of the vasodilating agent acetylcholine (ACh). In the concentration–response curve, aortic relaxation data are expressed as a percentage of the maximal contraction induced by the pre-contracting agent PE for each aortic segment. By normalizing ACh-induced relaxation against the PE-induced maximal contraction response for each aortic segment, we ensured that the observed differences in ACh-induced relaxation are not merely a result of inherent differences in the initial PE response, but rather a correct measure of the specific response to ACh stimulation. In both PE and ACh dose–response curves, the maximum contraction or relaxation responses achieved by control aortic rings were set at 100% arbitrarily, and the remaining experimental groups were compared to controls. To evaluate the effects of NO during smooth muscle contraction, aortic segments were pre-incubated with a non-selective NOS inhibitor, L-arginine methyl ester hydrochloride (L-NAME, 200 µM), prior to PE application. To evaluate the effects of NO originating from iNOS activation, aortic rings were pre-incubated with an irreversible and specific iNOS inhibitor, 1400 W (1 µM), prior to PE application.

### 4.7. Measurements of Aortic Diameters and Rupture Points

To assess the internal lumen diameter of mouse aorta, 2 mm aortic sections from the region adjacent to the aneurysm area were harvested and anchored onto the myograph chambers between two pins as described previously [87]. In the myograph chamber, the distance between the two pins (on which the aortic ring is anchored) at the initial point, where the generated force is first recorded (when the aortic wall initially touches the pin), is an indicator of the aortic luminal diameter in the resting condition. In another set of experiments, we tested the wall strength of the aortic segments by gradually increasing the distance between the two myograph pins (200 µm diameter) in 100 µm intervals, which correlates with an increase in the estimated length of vascular smooth muscle within the vessel wall. In our experiments, the stretching protocol was standardized, with each step lasting approximately 30 s to ensure consistency across samples. This time was chosen to minimize the influence of wall viscosity on the results. The stretching steps were repeated until the aortic segment could not maintain its tone, and the force (stress point) at which the anchored aortic segment ruptured was recorded as the rupture point, as previously described [87].

### 4.8. Aortic Wall Elastin Staining

The aortic arch of each mouse was collected, carefully cleaned, and fixed in 10% buffered formalin for 24 h and then transferred into 70% ethanol to be used for histological studies. Samples were sent to HistoWiz (Brooklyn, NY, USA) for processing, embedding, sectioning, and Verhoeff-van Gieson staining. To ensure consistent imaging, all stained slides were imaged, using an Olympus light microscope and Zeiss AXIO digital camera (Olympus American Inc., Center Valley, PA, USA), by an independent investigator blinded to genotype. Verhoeff-van Gieson staining was used to analyze medial elastin structure in serial cross sections (10 µm) of the aortic arch after rehydration of tissue sections. Elastin fragmentation within the aortic wall sections was examined using ImageJ Version 1.51 (Media Cybernetics, Bethesda, MA, USA) object tracing tools by tracing elastin fibers measured in pixels and converted to micrometers. Processing, staining, and analysis were blinded to prevent bias.

### 4.9. Statistical Analysis

Small vessel chamber myography experiments and data analysis were performed by a single investigator blinded to animal genotypes and treatment. Histological staining and images were processed by HistoWiz (Brooklyn, NY, USA), blinded to genotype and treatment. Image and data analysis were also performed by an independent lab member blinded to animal genotypes and treatment. Statistical analysis and construction of all concentration–response curves and graphs were performed using GraphPad Prism 10.2.3 Software. In all bar graph data, to compare PE-induced vasoconstriction, we have expressed all values as a percentage of the PE response relative to the prior peak KCl response for each aortic ring. Similarly, for the comparison of ACh-induced vasorelaxation, all values are presented as a percentage of the ACh response relative to the prior peak PE response for each aortic ring.

We also performed the normality tests for control and MFS mice aortic data using the Shapiro–Wilk test. The Shapiro–Wilk test showed that the data were normally distributed (W = 0.9844, *p* = 0.9273). Since the *p*-value was greater than 0.05, we concluded that the data did not significantly deviate from a normal distribution, and therefore the use of ANOVA was justified. A two-way analysis of variance (ANOVA) test was used to compare three or more groups, followed by a post hoc Tukey test for multiple statistical comparisons. Data are presented as Mean ± SE, where a *p*-value less than 0.05 (*p* < 0.05) is considered significant.

## 5. Conclusions

In conclusion, this report is the first investigation of the potential regulatory role of Cav1 protein in the mouse model of MFS with a focus on the impact on endothelial and smooth muscle function (Figure 10). The study of Cav1 in the context of MFS-associated aortic aneurysms is not only important for a deeper understanding of the disease’s pathophysiology, but also holds promise for innovative treatments. Our findings on Cav1’s regulation of NO production and endothelial function suggest promising avenues for complementary therapies. Specifically, targeting the Cav1-NO pathway in male MFS patients, where Cav1 ablation improved aortic wall strength, could increase NO availability and improve vascular function. In contrast, the lack of similar benefits in female MFS mice underscores the need for sex-specific treatment strategies. Estrogen’s inhibitory effects on Cav1 activity can likely lead to higher baseline NO production in females, potentially diminishing the impact of *Cav1KO*. This suggests that estrogen-mediated NO production may overshadow the effects of *Cav1KO*, whereas, in males, *Cav1KO* significantly boosts NO levels, contributing to improved aortic wall strength. By bridging the gap between molecular insights and clinical applications, future research on Cav1 could pave the way for more effective, sex-specific management strategies for MFS, ultimately improving patient outcomes.

## Data Availability

The data presented in this study will be available on request from the corresponding author.
